# Dietary intake of patients with moderate to severe COPD in relation to fat-free mass index: a cross-sectional study

**DOI:** 10.1186/s12937-015-0020-5

**Published:** 2015-04-10

**Authors:** Damla Yılmaz, Nermin Çapan, Sema Canbakan, Halit Tanju Besler

**Affiliations:** 1 Faculty of Health Sciences, Department of Nutrition and Dietetics, Hacettepe University, Ankara, Turkey; 2 Department of Respiratory Medicine, Atatürk Chest Diseases and Thoracic Surgery Training and Research Hospital, Ankara, Turkey

**Keywords:** COPD, Nutritional status, Dietary intake, Body composition, Fat-free mass

## Abstract

**Background:**

Fat-free mass (FFM) depletion has been shown to be a better predictor of mortality than BMI in chronic obstructive pulmonary disease (COPD) patients. The specific aim of the current study was to assess the nutritional status of stable COPD patients in relation to fat free mass index profiles.

**Methods:**

We investigated 65 male moderate-to-severe stable COPD patients. A self-reported questionnaire was applied about general characteristics and smoking history. Nutritional intake was assessed by using a 54–item quantitative food frequency questionnaire. Weight, height, mid-upper arm circumference (MUAC), waist circumference (WC), handgrip strength and body composition measurements were taken by a trained dietitian. The data were analyzed with SPSS 15.0 software.

**Results:**

The mean age of the patients was 62.1 ± 8.9 years. Among all of the patients 13.8% was underweight (BMI < 21 kg/m^2^) and 18.5% had a low fat-free mass index (FFMI < 16 kg/m^2^). The percentages of the patients who did not meet the daily recommended intakes (RNI) were highest for magnesium (93.8%) and calcium (92.3%). Mean daily consumptions of milk-yogurt, red meat and fruits were significantly low in the low FFMI group compared to normal FFMI group (for all; p < 0.05). Patients with normal FFMI had significantly higher weight, height, WC, MUAC, handgrip strength, fat and fat-free mass than the patients with low FFMI (for all; p < 0.05).

**Conclusions:**

Dieticians should be aware of COPD patients with low FFMI in order to evaluate the nutritional intake and therefore plan nutritional strategies to improve prognosis of the disease.

## Background

Chronic Obstructive Pulmonary Disease (COPD) is a preventable disease characterized by persistent airflow limitation that is usually progressive. It is a leading cause of morbidity and mortality worldwide and results in an economic and social burden [1]. COPD should not be considered as a localized pulmonary disorder but as a systemic disease. Well-characterized systemic features are muscle atrophy and weakness and osteoporosis [2].

The nutritional status of patients with COPD has been considered an important factor that influences the prognosis of the disease [3]. Approximately 20–40% of COPD outpatients have been reported as underweight or malnourished [4]. Body composition is reported to be one of the main determinants of functional disability of COPD patients independent of respiratory functions [5]. Weight loss and depletion of fat-free mass (FFM) may be observed in stable COPD patients, irrespective of the degree of airflow limitation and they are reported to contribute to morbidity, disability, and handicap [6]. Increased muscle protein break-down is a key feature in muscle wasting. This process of cachexia can be considered the result of interplay of systemic factors, including systemic inflammation, oxidative stress, and growth factors that may synergize with local factors leading to protein imbalance [7]. It is important to recognize that muscle mass may be reduced in COPD patients despite a normal BMI [8]. Fat-free mass index (FFMI) has been reported to provide information beyond that provided by body mass index (BMI) [9,10]. It has been shown that fat-free mass (FFM) depletion is a better predictor of mortality than BMI alone in COPD patients [9]. A recent study on 564 moderate to severe COPD patients in the Netherlands showed that disturbances in body composition were associated with dramatic differences in macro- and micronutrient intake [11].

Therefore, the specific aims of the current study were to examine the nutritional status of stable moderate to severe male COPD patients and to evaluate energy, macro- and micronutrient intakes in relation to fat free mass index profiles.

## Methods

### Design and participants

A cross-sectional study was conducted to determine the nutritional status of male COPD patients. The study population was recruited from COPD patients who visited the outpatient respiratory clinics of Ankara Ataturk Chest Diseases and Thoracic Surgery Training and Research Hospital, Turkey. Patients who are over 45 years of age, without any signs of infection and cognitively intact to answer the questions were invited to the study. The diagnosis and staging of COPD was made by pulmonologists according to American Thoracic Society/European Respiratory Society (ATS/ERS) and Global Initiative for Chronic Obstructive Lung Disease (GOLD) guidelines [1,6]. To be included in the study, patients had to be in a stable condition and not have reported acute symptoms or therapy modifications in the 30 days before enrollment. Patients with chronic kidney failure, diabetes, malignancy, congestive heart failure, myocardial infarction in the last 2 years were excluded from the study. In total, 65 male COPD patients (33 moderate, 32 severe) were included in the final data analysis of this study. The study protocol complied with the principles laid down in the Declaration of Helsinki and was approved by the Hacettepe University Senate Ethics Committee (B.30.2. HAC.0.70.00.01/431-1855 2010). Written informed consent was obtained from all subjects.

### Measurements

#### Characteristics of participants

A self-reported questionnaire was applied to the patients by face-to-face interview method to collect information about age, gender, educational level, total monthly family income, duration since diagnosed with COPD and smoking history.

#### Nutritional intake and physical activity

Nutritional intake and habitual food consumption was assessed by an 54–item quantitative food frequency questionnaire asking for dietary habits in the last 6 months by a trained dietitian. In order to assist respondents in identifying the actual quantity of the foods, a Turkish food photograph catalogue was used. Reported information was converted into a daily intake frequency of each item, which was in turn converted into the daily intake in grams per day for each food. The dietary data was analysed using BeBIS-6.1 (Nutrition Information Systems Software) and total intake of energy, carbohydrate, protein, fat, fiber, vitamin A, vitamin E, vitamin C, vitamin B12, calcium, iron, zinc and magnesium were calculated. Dietary intake was individually compared with gender- and age group- specific Turkish recommendations given in the Dietary Guidelines for Turkey (DGT) [12]. The exact RNI values were used as strict cutoff values to categorize dietary intake (e.g., <RNI and ≥ RNI). The physical activity levels (PAL) were assessed by a 24-h physical activity recall by a trained dietitian.

#### Anthropometric measurements and body composition

Anthropometric assessment consisted of determination of weight (kg), height (m), mid-upper arm circumference (MUAC) (cm) and waist circumference (cm). Height was measured with a clinical stadiometer while the patient was standing barefoot. Body weight was measured using a calibrated scale, with the patient wearing light clothes and no shoes. BMI (kg/m^2^) was calculated as weight/height^2^ and was classified according to European Respiratory Society (ERS) and American Thoracic Society’s (ATS) recommendation [6]. Mid-upper arm circumference was measured on the non-dominant arm using a non-stretch tape measure at the mid point between acromion and olecranon. Waist circumference (WC) measurement was made around patient’s bare midriff, at the midpoint between the lowest rib and the iliac crest, at the end of gentle expiration while standing without shoes with a non-stretch tape.

Handgrip strength was assessed as a measure of peripheral skeletal muscle strength. A digital handgrip-dynamometer (Takei TKK-5401) was used to determine the isometric grasp in each hand by measuring the maximally developed strength of the flexors of the fingers. Two measurements were made in each hand with the arm unsupported. The mean value of left and right hand strength was used for statistical analysis.

Body composition was assessed using bioelectrical impedance analysis (BIA) (Bodystat 1500; Bodystat Ltd, Douglas, UK) with subjects lying supine, with four surface electrodes placed on the right wrist and ankle. Measurements were obtained in the morning after a fast of at least 3 hours and urination 30 minutes prior to the procedure. The fat-free mass index (FFMI) (kg/m^2^) was calculated as the ratio of FFM to height in meters squared. Fat free mass index was classified as low FFMI (<16 kg/m^2^) and normal FFMI (≥16 kg/m^2^) [8]. All of the anthropometric measurements were performed by a trained dietitian.

#### Spirometry and disease severity

Spirometric evaluation of the patients was performed using a computerized spirometer (Spirolab III SFT) by spirometry technicians. Spiromeric values of the forced expiratory volume in the first second (FEV_1_), forced vital capacity (FVC), and FEV_1_/FVC were recorded as percent predicted values. The FEV_1_% predicted was measured with the highest value from at least three technically acceptable spirometric manoevres being used. Classification of disease stage was based on the forced expiratory volume in the first second (FEV_1_) value as per GOLD guideline i.e. stage I FEV1 ≥ 80%predicted, stage II 50% ≤ FEV1 < 80%predicted, stage III 30% ≤ FEV1 < 50%predicted and stage IV < 30% of predicted [1].

#### Blood parameters

Serum sample from each subject was obtained by a trained nurse at a fasting state of ≥8 hours and they were analyzed for visceral protein stores represented by total protein and serum albumin. The measurements were performed by the clinical chemistry laboratory of Ankara Ataturk Chest Diseases and Thoracic Surgery Training and Research Hospital with the Mannheim/Hitachi 747 analyzer using the bromcresol green method (Roche, Oxford, CT).

### Data analysis

The data were analyzed with SPSS (Statistical Package for the Social Sciences) WIN 15.0. Descriptive statistics reported frequencies, percentages, mean (±SD), minimum and maximum values where appropriate as well as chi-square or Fisher differences. The normality of the data distribution was tested using visual (histogram and probability graphics) and analytical methods (Kolmogrov-Smirnov/Shapiro-Wilk tests). Student’s *t* test (under parametric conditions) or Mann–Whitney U test (under non-parametric conditions) for unpaired data was used for comparison of general characteristics, nutritional and anthropometric data between the low FFMI and normal FFMI groups. Spearman correlation test examined the relationship between FFMI (kg/m^2^) and age, spirometry, handgript strength, total energy expenditure (TEH), physical activity level (PAL), serum albumin and total protein levels.

## Results

### General characteristics

**Figure 1:**
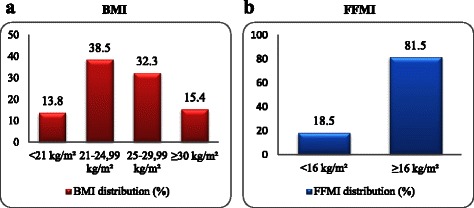
**BMI and FFMI distributions of COPD patients (n = 65). a**. Values indicating the patients with BMI values <21 kg/m^2^ (underweight), between 21-24.99 kg/m^2^ (normal weight), between 25-29.99 kg/m^2^ (overweight) and ≥30 kg/m^2^ (obese) are shown as percentages (%). **b**. Values indicating the patients with FFMI values <16 kg/m^2^ (low FFMI) and ≥16 kg/m^2^ (normal FFMI) are shown as percentages (%). Abbreviations: BMI: Body mass index; FFMI: Fat-free mass index.

**Figure 2:**
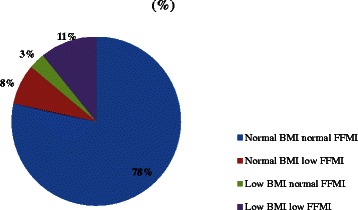
**The prevalence of different depletion categories.** Blue: normal BMI and FFMI; red: normal BMI and FFM depletion; green: low BMI and normal FFM; purple: low BMI and FFMI.

The mean age of the patients (n = 65) was 62.1 ± 8.9 years and most were retired (81.5%) and married (90.8%). The mean FEV_1_% predicted was 50.2 ± 13.6%. Duration since diagnosed with COPD was significantly lower in moderate patients than severe patients (5.6 ± 8.7 and 8.2 ± 9.6 years respectively; p = 0.043). Mean age for starting to smoke was 14.9 ± 4.4 years and the cumulative smoking was 53.5 ± 28.8 pack.years (Table 1). All participants had health insurance and 60% of the patients reported a monthly family income of less than 700 Turkish Liras (≈US$314). Distributions of BMI classification of the patients according to the classification of European Respiratory Society and American Thoracic Society (ERS/ATS) and FFMI are given in Figure 1. Among all of the patients 13.8% was underweight, 32.3% was overweight and 15.4% was obese. On the other hand, low FFMI was assessed in the 18.5% of the patients. While 11% of the patients had both low BMI and FFMI, 8% of the patients had a low FFMI despite a normal BMI (Figure 2). Moderate and severe COPD patients did not show significant difference in context of BMI and FFMI distributions when evaluated separately (data not shown).

**Table 1:** **General characteristics of COPD patients (n = 65)**

Characteristics	
Age (y)	62.1 ± 8.9
45-64	38 (58.5)
≥65	27 (41.5)
FEV_1_ (% of predicted)	50.2 ± 13.6
FVC (%)	64.5 ± 16.6
FEV_1_/FVC	61.4 ± 7.7
Smoking	
Initiation age (y)	14.9 ± 4.4
Pack.years	53.5 ± 28.8
Duration of COPD (y)	6.9 ± 9.2
BMI (kg/m^2^)	25.3 ± 4.3
FFMI (kg/m^2^)	18.9 ± 2.6

### Nutritional intake and habitual food consumption

Table 2 lists the participants’ nutritional intake status assessed by quantitative food frequency questionnaire. The mean total energy intake was 1906.3 ± 567.4 kcal and did not show significant difference between low FFMI and normal FFMI groups (p = 0.478). Seventy five percent of the patients’ energy intakes were not meeting the recommended daily intake levels. Although the difference was not statistically significant, the frequency of patients whose dietary energy intake did not meet the RNI levels was higher in the low FFMI group (91.7%).

**Table 2:** **Energy and nutrient intakes of COPD patients assessed by food frequency questionnaire**

	Low FFMI (n = 12)	Normal FFMI (n = 53)		Total (n = 65)
	X ± SD	X ± SD	p	X ± SD
Energy (kcal)	1770.0 ± 338.2	1937.2 ± 605.7	0.478	1906.3 ± 567.4
RNI (%)	81.3 ± 15.1	86.1 ± 24.4	0.519	85.2 ± 22.9
<RNI	11 (91.7)	38 (71.7)	0.266	49 (75.4)
Carbohydrate (g)	258.4 ± 46.9	276.6 ± 92.3	0.488	273.2 ± 85.7
Protein (g)	68.4 ± 15.4	73.7 ± 26.7	0.946	72.7 ± 25.0
Fat (g)	48.1 ± 13.6	55.5 ± 21.5	0.398	54.1 ± 20.4
Ratio of C:P:F	60:16:24	58:16:26	-	58:16:26
Fiber (g)	19.3 ± 3.8	21.0 ± 6.9	0.499	20.7 ± 6.5
RNI (%)	66.4 ± 13.2	72.3 ± 23.9	0.254	71.2 ± 22.3
<RNI	12 (100.0)	47 (88.7)	0.583	59 (90.8)
Vitamin A (mcg)	1029.2 ± 1365.4	782.2 ± 621.6	0.710	827.8 ± 802.3
RNI (%)	114.4 ± 151.2	86.9 ± 69.1	0.340	92.0 ± 89.1
<RNI	8 (66.7)	41 (77.4)	0.470	49 (75.4)
Vitamin E (mg)	16.3 ± 5.1	17.3 ± 7.0	0.710	17.1 ± 6.6
RNI (%)	108.5 ± 33.7	115.6 ± 46.5	0.618	114.3 ± 44.3
<RNI	4 (33.3)	20 (37.7)	1.000	24 (36.9)
Vitamin B12 (mcg)	4.9 ± 4.8	3.8 ± 3.0	0.442	4.0 ± 3.4
RNI (%)	202.1 ± 201.1	160.0 ± 123.3	0.350	167.7 ± 139.9
<RNI	3 (25.0)	21 (39.6)	0.511	24 (39.6)
Vitamin C (mg)	115.2 ± 34.7	115.6 ± 60.3	0.389	115.5 ± 56.2
RNI (%)	128.0 ± 38.6	128.5 ± 67.0	0.979	128.4 ± 62.4
<RNI	1 (8.3)	20 (37.7)	0.084	21 (32.3)
Calcium (mg)	654.0 ± 141.7	759.8 ± 334.8	0.302	740.2 ± 310.2
RNI (%)	54.5 ± 11.8	65.2 ± 31.4	0.057	63.2 ± 29.1
<RNI	12 (100.0)	48 (90.6)	0.575	60 (92.3)
Iron (mg)	9.1 ± 1.9	9.9 ± 3.3	0.594	9.8 ± 3.1
RNI (%)	91.2 ± 19.2	99.2 ± 33.1	0.423	97.7 ± 31.0
<RNI	7 (58.3)	30 (56.6)	0.913	37 (56.9)
Zinc (mg)	8.1 ± 1.7	9.0 ± 3.2	0.660	8.8 ± 3.0
RNI (%)	74.0 ± 15.8	81.8 ± 29.2	0.375	80.3 ± 27.3
<RNI	12 (100.0)	42 (79.2)	0.109	54 (83.1)
Magnesium (mg)	205.3 ± 53.8	247.0 ± 94.2	0.217	239.3 ± 89.3
RNI (%)	48.9 ± 12.8	58.8 ± 22.4	0.145	57.0 ± 21.3
<RNI	12 (100.0)	49 (92.5)	1.000	61 (93.8)

Energy and nutrient intakes of COPD patients assessed by food frequency questionnaire. Values for actual nutrient intakes and percentage of meeting the RNI levels are shown as mean ± SD. Values indicating the number of patients with nutrient intakes below RNI (<RNI) are shown as number and percentage (%) in parenthesis. None of the differences between the low FFMI group and the normal FFMI group were significant. RNI: recommended nutrient intake.

Daily intakes of carbohydrate, protein, fat and fiber were 273.2 ± 85.7 g, 72.7 ± 25.0 g, 54.1 ± 20.4 g and 20.7 ± 6.5 g respectively. Mean fiber intake was 20.7 ± 6.5 g and 90.8% of the patients could not meet RNI for fiber. Neither vitamin nor mineral intakes were different between the two FFMI groups. The mean percentages of meeting the daily recommended intakes (RNI) were lowest for magnesium (57.0 ± 21.3%) and calcium (62.2 ± 29.1%). None of the patients with low FFMI could meet the RNI levels for fiber, magnesium, calcium and zinc.

Habitual daily food consumption of COPD patients assessed by quantitative food frequency questionnaire is given in Table 3. Mean daily consumption of dairy products was 220.1 ± 170.4 g and 95.4% of the patients’ dairy consumption was below the recommended levels. Mean consumption of legumes among COPD patients was 8.9 ± 5.6 g/day and nearly 97% was not meeting the RNI for legumes. Mean daily fruit and vegetable consumption was 389.9 ± 200.1 g and 92.3% of the patients’ consumption was below the RNI for fruits and vegetables. Mean daily consumptions of milk-yogurt, red meat and fruits were found to be significantly low in the low FFMI group compared to normal FFMI group (for all; p < 0.05).

**Table 3:** **Daily food consumption of COPD patients assessed by quantitative food frequency questionnaire (n = 65)**

	Low FFMI (n = 12)	Normal FFMI (n = 53)		Total (n = 65)
Food groups (g/day)	X ± SD	X ± SD	p	X ± SD
Dairy products	117.9 ± 86.6	243.3 ± 176.7	0.007*	220.1 ± 170.4
<RNI	12 (100.0)	50 (94.3)	1.000	62 (95.4)
Milk-yogurt	85.4 ± 86.4	208.1 ± 174.0	0.004*	185.5 ± 167.9
<RNI	12 (100.0)	51 (96.2)	1.000	63 (96.9)
Cheese	32.5 ± 19.9	35.2 ± 22.9	0.986	34.7 ± 22.2
<RNI	3 (25.0)	17 (85.0)	0.741	20 (30.8)
Meats, eggs, legumes, nuts	110.7 ± 43.8	123.2 ± 62.1	0.800	120.9 ± 59.0
<RNI	9 (75.0)	36 (67.9)	0.673	55 (84.6)
Red meat	4.8 ± 6.5	12.7 ± 14.3	0.044*	11.2 ± 13.5
Chicken, turkey	33.6 ± 31.6	44.7 ± 34.2	0.260	42.7 ± 33.8
Fish	23.0 ± 26.9	28.9 ± 27.1	0.450	27.8 ± 26.9
Eggs	27.1 ± 14.2	20.7 ± 17.6	0.116	21.9 ± 17.1
<RNI	0 (0.0)	8 (15.1)	0.333	8 (12.3)
Legumes	8.2 ± 4.8	9.1 ± 5.8	0.428	8.9 ± 5.6
<RNI	12 (100.0)	51 (96.2)	1.000	63 (96.9)
Nuts	14.1 ± 15.5	7.2 ± 11.1	0.531	8.5 ± 12.2
Bread-grains	310.9 ± 112.3	343.4 ± 140.1	0.434	337.3 ± 135.1
<RNI	10 (83.3)	40 (75.5)	0.717	38 (58.5)
Fruits	124.0 ± 109.3	222.3 ± 121.8	0.013*	204.1 ± 124.8
Vegetables	167.5 ± 104.1	190.0 ± 129.0	0.576	185.8 ± 124.3
Fruits and vegetables	150.3 ± 43.4	412.2 ± 204.4	0.058	389.9 ± 200.1
<RNI	12 (100.0)	48 (90.6)	0.575	60 (92.3)
Oils-fats	30.3 ± 20.2	26.8 ± 11.2	0.906	27.4 ± 13.2
<RNI	8 (66.7)	42 (79.2)	0.449	50 (76.9)
Sugar	41.6 ± 30.5	42.5 ± 36.5	0.946	42.3 ± 35.3
<RNI	10 (83.3)	36 (67.9)	0.484	50 (76.9)

Daily food consumption of COPD patients assessed by quantitative food frequency questionnaire (n = 65). Values for actual intakes are shown as mean ± SD. Values indicating the number of patients with food consumption below RNI (<RNI) are shown as number and percentage (%) in parenthesis. Asterisks indicate a significant difference between groups: *P < 0.05. Daily dairy, milk-yogurt, red meat and fruit consumptions of patients with low FFMI were significantly lower. RNI: recommended nutrient intake.

### Anthropometric measurements, body composition, physical activity level and blood parameters

Anthropometric measurements, handgrip strength, total energy expenditure (TEE), physical activity level (PAL) and some blood parameters of COPD patients are given in Table 4. Patients with normal FFMI had significantly higher weight, height, WC, MUAC, fat and fat-free mass than the patients with low FFMI. Mean handgript strength of the low FFMI group was 26.1 ± 4.7 kg while it was 34.6 ± 7.9 kg in the normal FFMI group (p = 0.001). Total energy expenditure (TEE) assessed by 24-h recall was also significantly low in the low FFMI group, but the physical activity levels (PAL) were similar. There was a positive weak correlation between FFMI and handgript strength (r = 0.331, p = 0.007) and a positive moderate correlation between FFMI and TEE (r = 0.548, p = 0.000) (Table 5). Hemoglobin and hemotoctrit levels were significantly lower in the low FFMI group (for all; p < 0.05) (Table 4). A positive weak correlation was found between FFMI and serum albumin (r = 0.288, p = 0.022) (Table 5).

**Table 4:** **Body composition, handgrip strength, physical activity level (PAL) and some blood parameters of COPD patients**

	Low FFMI (n = 12)	Normal FFMI (n = 53)		Total (n = 65)
Characteristics	X ± SD	X ± SD	p	X ± SD
Weight (kg)	55.1 ± 6.4	75.4 ± 10.9	0.000**	71.6 ± 12.9
Height (cm)	164.1 ± 5.9	168.8 ± 5.9	0.015*	167.9 ± 6.1
WC (cm)	83.9 ± 7.6	101.0 ± 11.4	0.000**	97.8 ± 12.6
MUAC (cm)	25.1 ± 1.7	30.2 ± 3.0	0.000**	29.3 ± 3.4
FM (g)	14.8 ± 3.4	18.8 ± 6.4	0.042*	18.1 ± 6.1
FM (%)	26.7 ± 3.6	24.5 ± 5.5	0.155	24.9 ± 5.2
FFM (g)	40.3 ± 4.0	56.3 ± 6.3	0.000**	53.4 ± 8.6
FFM (%)	73.3 ± 3.6	75.5 ± 5.5	0.146	75.1 ± 5.2
Handgript strength (kg)	26.1 ± 4.7	34.6 ± 7.9	0.001*	33.1 ± 8.1
TEE (kkal/d)	2031.9 ± 344.3	2463.5 ± 426.6	0.002*	2382.8 ± 443.5
PAL	1.5 ± 0.2	1.6 ± 0.2	0.156	1.5 ± 0.2
WBC	8.3 ± 2.2	8.4 ± 1.8	0.882	8.4 ± 1.9
HG (g/dL)	14.5 ± 1.5	15.4 ± 1.3	0.038*	15.3 ± 1.4
HCT (%)	43.4 ± 4.3	46.3 ± 3.8	0.024*	45.7 ± 4.0
Serum albumin (g/dL)	4.1 ± 0.4	4.3 ± 0.3	0.051	4.2 ± 0.3
Serum total protein (g/dL)	6.8 ± 0.4	7.0 ± 0.5	0.289	7.0 ± 0.5

Body composition, handgrip strength, physical activity level (PAL) and some blood parameters of COPD patients. Values are shown as mean ± SD. Asterisks indicate a significant difference between groups: *P < 0.05; **P < 0.001. Patients with low FFMI had significantly lower weight, height, WC, MUAC, FM, FFM, handgrip strength, TEE, HG and HCT values. *Abbreviations: WC* waist circumference, *MUAC* mid-upper arm circumference, *FM* fat mass, *FFM* fat-free mass, *TEE* total energy expenditure, *PAL* physical activity level, *WBC* white blood cells, *HG* hemglobin, *HCT* hematoctrit.

**Table 5:** **Spearman’s rank correlation between FFMI (kg/m** ^**2**^ **) and some parameters**

	FFMI (kg/m ^ 2 ^ )	
	r	p
Age	−0.059	0.641
FEV_1_ (%)	0.092	0.465
FEV_1_/FVC (%)	0.217	0.082
Handgript strength (kg)	0.331	0.007*
TEE (kkal/d)	0.548	0.000**
PAL	0.216	0.084
Serum albumin (g/dL)	0.288	0.022*
Serum total protein (g/dL)	0.159	0.213

Spearman’s rank correlation between FFMI (kg/m^2^) and some parameters. Asterisks indicate a significant correlation between groups: *P < 0.05; **P < 0.001.

## Discussion

### General characteristics

The present study which included male COPD patients with moderate to severe airway obstruction showed that dietary intake and habitual food consumption differ in relation to fat-free mass index. The link between aging and the pathogenesis of COPD is strongly supported [10] and longer duration of the disease since diagnose in severe COPD patients in our study can be explained by the progressive nature of the disease [1]. Cigarette smoking is the most important environmental risk factor for the development of COPD [13]. In our study the mean age for starting to smoke corresponded to adolescence, similar to the findings of a recent study by Kim et al. [14], who found the mean initiation age of smoking of COPD patients as 16.4 ± 4.8 years.

In the current study 18.5% of patients were characterized by a low FFMI. Similarly, a study among 72 COPD outpatients suffering from a moderate degree of airflow obstruction showed that prevalance of fat-free mass depletion was 18% in male patients [15]. In another study, among moderate to severe COPD patients the prevalance of fat free mass depletion was 20.3% in male patients [16]. A recent study by Van de Bool et al. [11] showed that almost 25% of moderate to severe COPD patients who were eligible for pulmonary rehabilitation were characterized by a low FFMI. These little discrepancies may be explained by different inclusion criteria of the studies. On the other hand, in a cross-sectional study among male COPD patients, depletion of fat-free mass based on fat-free mass index was about 42% [17]. This discrepancy can be due to more severe COPD being present in the study as they mentioned that the prevalance of low FFMI was high among severe to very severe (stage IV) COPD patients.

Increased BMI does not protect against fat-free mass depletion in COPD, since there is a preferential loss of muscle tissue in this disease [18]. In our study 8% of the patients had low FFMI despite a BMI ≥21 kg/m^2^. A study by Pirabbasi et al. [17] showed that among 271 male COPD patients 11.1% had a low FFMI (<16 kg/m^2^) despite a normal BMI (≥21 kg/m^2^). In the study by Vermeeren et al. [16], the prevalence of being underweight was 17% whereas prevalence of FFM depletion was 42%. It should be noted however that in their study they used <18.5 kg/m^2^ as the BMI cutoff for being underweight.

### Nutritional intake and habitual food consumption

In the current study, mean daily energy intakes of COPD patients was 1906 kcal similarly to a group of 275 moderate to severe COPD patients (93% male) in Spain, whose mean daily energy intake was 2033 kcal [19]. According to Turkey Health and Nutrition Survey-2010, mean energy intake of Turkish individuals was 1918 kcal in the 51–64 years age group and 1706 kcal in the 65–74 year age group [20]. A study in Malaysia with 149 COPD patients showed that dietary energy intake of patients (assessed by one day or two days record) was below Malaysian RNI [18]. Compared to the Malaysian sample, in our study prevalance of patients with energy intake below RNI is lower yet clinically significant (93% vs 75%, respectively). A recent study by Van de Bool et al., which is the only study to evaluate dietary intake of COPD patients in relation to body composition until now, showed that COPD patients with low FFMI reported higher energy intake than patients with normal FFMI [11]. On the contrary, in the current study mean energy intake of patients in the low FFMI group was lower than normal FFMI group (1770 ± 338 and 1937 ± 606 kcal, respectively).

Selective wasting of fat-free mass is suggesting a disturbed protein balance in COPD patients [21]; hence, protein intake of depleted COPD patients is recommended to exceed 1.5 g/kg/day [22]. Increased protein intake and physical activity, in the form of resistance training, stimulate muscle protein synthesis in the elderly [23]. In the study of Van de Bool et al. [11], COPD pateints with low FFMI reported significantly higher protein intake per kg body weight. In the current study, protein intake of patients did not differ between low and normal FFMI groups. However, daily consumptions of dairy products and red meat were significantly low in the low FFMI group. This finding is considerable since high-quality protein sources such as whey protein, milk, and beef have been shown to improve protein synthetic response in the elderly [24].

In the current study, majority of patients’ daily milk and yogurt consumption was below RNI and this finding was more marked in the patients with low FFMI. Milk proteins (casein and whey) are known for their high branched-chain amino acid (BCAA) content, which include leucine (LEU), isoleucine (ILE) and valine (VAL) [25]. Since skeletal muscle is a major site of (BCAA) catabolism in disease state, they are used for maintenance of protein quality and repair process of tissues [26]. Plasma levels of BCAAs, particularly leucine, are reduced in patients with COPD [27] and a significant association was found between low levels of BCAAs and depletion of FFM [17]. Engelen et al. showed an elevated anabolic response to sip feeding of a casein protein meal in patients with COPD [28]. All of these findings make milk proteins an important preventive approach to conserve muscle mass in COPD.

In this study, a vast majority (92%) of the COPD patients could not meet RNI for fruits and vegetables with mean daily consumption of fruits being significantly lower in the low FFMI group compared to normal FFMI group. This finding is concerning since cross-sectional studies have showed a significant positive association between fruit and vegetable (FV) intake and forced expiratory volume in 1 s (FEV_1_), with stronger evidence for fruit consumption [29,30]. Data from the MORGEN study showed that higher intakes of antioxidants such as vitamin C, beta-carotene and flavonoids are associated with higher FEV_1_ values, compared with low intakes [31,32]. Moreover, Walda et al. [33] demonstrated an inverse association between fruit intake and 20 yr COPD mortality.

The major deficiencies were assessed in magnesium and calcium intakes in the current study. Mean magnesium intake of COPD patients was 239.3 ± 89.3 mg in our study. According to National Turkish Health and Nutrition Survey (NTHNS) 2010, mean magnesium intake was 290.8 mg in the 51–64 year old group, 271.3 mg in the 65–74 year old group and 241.7 mg in 75 years and older [20]. Mean magnesium intake of COPD patients in our study was lower than all of the age groups in the national survey. Low consumption of dark leafy vegetables, nuts and seeds due to chewing problems [34], or legumes due to gastrointestinal disturbances [35] might be the reason of low magnesium intake in our group of COPD patients.

Mean calcium intake in the current study was 740.2 ± 310.2 mg and 92% of the patients’ intake could not meet RNI. Calcium intake in the NTHNS-2010 was 712.7 mg in the 51–64 year olds, 677.2 mg in the 65–74 year olds and 592.6 mg in 75 years and older [20]. In the study by Van de Bool et al. which evaluated the dietary intake of COPD patients assessed by using a cross-check dietary history in in Netherlands, calcium intake was reported as “too low” since 72% of the patients’ dietary intake could not meet the RNI [11]. In the COPD patients in this study, the reported percentage of patients with calcium intake below recommendations was remarkably higher. In a Spanish group of 275 moderate to severe COPD patients, prevalence of complience with recommendations was 31% for magnesium and 49% for calcium and similar to our study magnesium was the major mineral deficieny [19].

We did not find any significant differences in daily macro- and micronutrient intakes between patients with low FFMI and normal FFMI. Unlikely to our study, Van de Bool et al. recently showed that intakes of calcium and vitamin A in COPD patients with low FFMI were significantly higher [11]. The consumption of legumes, dairy products, fruits and vegetables were lowest as majority of the patients’ intakes did not comply with the recommendations in our sample. In a Spanish group of 275 moderate to severe COPD patients daily legume consumption was 31 ± 21 g while in our study it is 8.9 ± 5.6 g. This difference may be attributed to cultural differences between the countries.

### Anthropometric measurements, body composition and physical activity level

In the present study mean weight, height, waist circumference (WC), mid-upper arm circumference (MUAC), fat mass and fat-free mass were significantly lower in patients with low FFMI. Mid-upper arm circumference correlate with total muscle mass and is therefore used to predict changes in the protein nutritional status [36]. Accordingly, in the present study mean MUAC of patients with low FFMI was significantly low.

Patients with COPD have a significantly reduced duration, intensity, and counts of daily physical activity when compared to healthy control subjects [37]. Low fat-free mass has been shown to impair exercise performance in COPD patients [38]. A recent study by Andersson et al. [39] showed that COPD patients who were more physically active were characterized not only by better pulmonary function but also higher BMI and FFMI. In the current study, mean total daily energy expenditure of patients with low FFMI was significantly low. Additionally, mean physical activity levels was lower in the low FFMI group, but the difference was not statistically significant.

Serum albumin is synthesized in the liver and is a marker of nutritional status. Data suggest that low serum albumin is associated with low appendicular skeletal muscle mass in elderly women and men. Reduced protein metabolism with aging may occur concurrently in the liver and muscle causing similar decrements in both serum albumin and muscle mass [40]. Although in our study mean serum albumin levels were not markedly different between low and normal FFMI groups, there was a positive weak correlation between FFMI and serum albumin.

Cesari et al. showed in their study that hemoglobin levels were associated with muscle and fat mass changes, and that decreased muscular strength occured in the presence of anemia in individuals who were 65 years and older [41]. Similarly to these findings, in our study patients with low FFMI had significantly low hemoglobin and hematocrit levels.

### Limitations

Some shortcomings of the current study need to be considered. First, no healthy control group could be included in the present analyses in order to compare the nutritional intake between COPD patients and healthy subjects. Nevertheless, results were compared with general findings in general older Turkish adults from the Natonal Turkish Nutrition and Health Survey-2010 [20]. Second, loss of FFM seems to be more frequent in patients with emphysema-type COPD than in patients with chronic bronchitis. Unfortunately, we were unable to differentiate COPD subtypes in our study. Third, the assessment of dietary intake of fat might be underestimated in food frequency questionnaire. The food frequency method is generally applied in order to assess the quality of dietary intake because it is able to provide data about particular food groups. While there is concern that food frequency questionnaires can be prone to measurement error [42], they have been shown to identify similar patterns of diet as other dietary methods [43].

Fourth, in the current study physical activity was assessed by a 24-h recall questionnaire. It has been reported that COPD patients overestimate the time spent walking and underestimate time spent standing. Therefore, using a multisensor armband or an accelerometer to assess physical activity would be more reliable [37].

## Conclusions

Health proffesionals, especially dietitians should be aware of COPD patients with low FFMI in order to evaluate the nutritional intake and therefore plan nutritional strategies to improve prognosis of the disease. Dietary strategies to prevent fat-free mass loss in COPD patient should be further investigated.

## Abbreviations

ATS: American Thoracic Society
BCAA: Branched-chain amino acids
BIA: Bioelectrical impedance analysis
BMI: Body mass index
COPD: Chronic obstructive pulmonary disease
DGT: Dietary Guidelines for Turkey
ERS: European Respiratory Society
FEV_1_: Forced expiratory volume in the first second
FFM: Fat-free mass
FFMI: Fat-free mass index
FVC: Forced vital capacity
GOLD: Global Initiative for Chronic Obstructive Lung Disease
HCT: Hematoctrit
HG: Hemoglobin
ILE: Isoleucine
LEU: Leucine
MUAC: Mid-upper arm circumference
NTHNS: National Turkish Health and Nutrition Survey
PAL: Physical activity level
RNI: Recommended nutrient intakes
TEH: Total energy expenditure
VAL: Valine
WBC: White blood cells
WC: Waist circumference

## References

[1] Global Initiative for Chronic Obstructive Lung Disease. Global Strategy for the Diagnosis, Management and Prevention of Chronic Obstructive Pulmonary Disease. Revised 2011. [http://www.goldcopd.org/uploads/users/files/GOLD_Report_2011_Feb21.pdf]

[2] Patel AR, Hurst JR (2011). Extrapulmonary comorbidities in chronic obstructive pulmonary disease: state of the art. Expert Rev Respir Med.

[3] Ferreira IM, Brooks D, Lacasse Y, Goldstein RS, White J. Nutritional supplementation for stable chronic obstructive pulmonary disease. Cochrane Database Syst Rev. 2008. Published online Oct 8. doi:10.1002/14651858.CD000998.pub2.

[4] Raguso CA, Luthy C (2011). Nutritional status in chronic obstructive pulmonary disease: role of hypoxia. Nutrition.

[5] Eisner MD, Blanc PD, Sidney S, Yelin E, Lathon P, Katz PP (2007). Body composition and functional limitation in COPD. Respir Res.

[6] Celli BR, MacNeei W (2004). Standards for the diagnosis and treatment of patients with COPD: a summary of the ATS/ERS position paper. Eur Respir J.

[7] Debigaré R, Maltais F, Côté CH (2001). Peripheral muscle wasting in chronic obstructive pulmonary disease: clinical relevance and mechanisms. Am J Respir Crit Care Med.

[8] Schols SMWJ, Soeters PB, Dingemans AMC, Mostert R, Frantzen PJ, Wouters EFM (1993). Prevalence and characteristics of nutritional depletion in patients with stable COPD eligible for pulmonary rehabilitation. Am Rev Respir Dis.

[9] Schols AMWJ, Brokhuizen R, Weling-Scheepers CA, Wouters EF (2005). Body composition and mortality in chronic obstructive pulmonary disease. Am J Clin Nutr.

[10] Vestbo J, Prescott E, Almdal T, Dahl M, Nordestgaard BG, Andersen T (2006). Body mass, fat-free body mass, and prognosis in patients with chronic obstructive pulmonary disease from a random population sample. Am J Respir Crit Care Med.

[11] Van de Bool C, Mattijssen-Verdonschot C, van Melick PP, Spruit MA, Franssen FM, Wouters EF (2014). Quality of dietary intake in relation to body composition in patients with chronic obstructive pulmonary disease eligible for pulmonary rehabilitation. Eur J Clin Nutr.

[12] The Ministry of Health of Turkey. Dietary Guidelines for Turkey. 5th Edition; 2006 [http://beslenme.gov.tr/content/files/yayinlar/ingilizce_yayinlar/books/dietary_guidelines.pdf]

[13] Mannino DM, Buist AS (2007). Global burden of COPD: risk factors, prevalence, and future trends. Lancet.

[14] Kim DK, Hersh CP, Washko GR, Hokanson JE, Lynch DA, Newell JD (2011). Epidemiology, radiology, and genetics of nicotine dependence in COPD. Respir Res.

[15] Engelen MPKJ, Schols AMWJ, Baken WC, Wesseling GJ, Wouters EFM (1994). Nutritional depletion in relation to respiratory and peripheral skeletal muscle function in outpatients with COPD. Eur Respir J.

[16] Vermeeren MAP, Creutzberg EC, Schols AM, Postma DS, Pieters WR, Roldaan AC (2006). Prevalence of nutritional depletion in a large out-patient population of patients with COPD. Respir Med.

[17] Pirabbasi E, Najafiyan M, Cheraghi M, Shahar S, Manaf ZA, Rajab N, et al. Predictors’ Factors of Nutritional Status of Male Chronic Obstructive Pulmonary Disease Patients. ISRN Nursing. 2012, Article ID: 782626, doi:10.5402/2012/782626 10.5402/2012/782626 PMC3504379 23209935

[18] Schols AM, Deutz NE, Mostert R, Wouters EF (1993). Plasma amino acid levels in patients with chronic obstructive pulmonary disease. Monaldi Arch Chest Dis.

[19] De Batlle J, Romieu I, Anto JM, Mendez M, Rodriguez E, Balcells E (2009). Dietary habits of firstly admitted Spanish COPD patients. Respir Med.

[20] TNHS-2010. Turkey Nutrition and Health Survey (TBSA) 2010. Ministry of Health Headquarters of Health Reseaeches, Hacettepe University Faculty of Health Sciences Department of Nutrition and Dietetics, Ankara Numune Training and Research Hospital (2014). Final Report of the Findings of Nutritional Status and Nutritional Habits.

[21] Engelen M, Schols A, LAmers RJS, Wouters EFM (1999). Different patterns of chronic tissue wasting among patients with chronic obstructive pulmonary disease. Clin Nutr.

[22] Vermeeren MA, Schols AM, Wouters EF (1997). Effects of an acute exacerbation on nutritional and metabolic profile of patients with COPD. Eur Respir J.

[23] Starling RD, Ades PA, Poehlman ET (1999). Physical activity, protein intake, and appendicular skeletal muscle mass in older men. Am J Clin Nutr.

[24] Symons TB, Sheffield-Moore M, Wolfe RR, Paddon-Jones DA (2009). Moderate Serving of High-Quality Protein Maximally Stimulates Skeletal Muscle Protein Synthesis in Young and Elderly Subjects. J Am Diet Assoc.

[25] Katsanos CS, Kobayashi H, Sheffield-Moore M, Aarsland A, Wolfe RR (2006). A high proportion of leucine is required for optimal stimulation of the rate of muscle protein synthesis by essential amino acids in the elderly. Am J Physiol.

[26] Engelen MPKJ, De Castro CLN, Rutten EPA, Wouters EFM, Schols AMWJ (2012). Enhanced anabolic response to milk protein sip feeding in elderly subjects with COPD is associated with a reduced splanchnic extraction of multiple amino acids. Clin Nutr.

[27] Engelen MP, Wouters EF, Deutz NE, Menheere PP, Schols AM (2000). Factors contributing to alterations in skeletal muscle and plasma amino acid profiles in patients with chronic obstructive pulmonary disease. Am J Clin Nutr.

[28] Engelen MP, Rutten EP, De Castro CL, Wouters EF, Schols AM, Deutz NE (2005). Altered interorgan response to feeding in patients with chronic obstructive pulmonary disease. Am J Clin Nutr.

[29] Smit HA, Grievink L, Tabak C (1999). Dietary influences on chronic obstructive lung disease and asthma: a review of the epidemiological evidence. Proc Nutr Soc.

[30] Romieu I, Trenga C (2001). Diet and obstructive lung diseases. Epidemiol Rev.

[31] Grievink L, Smit HA, Ocke MC, van’t Veer P, Kromhout D (1998). Dietary intake of antioxidant (pro)-vitamins, respiratory symptoms and pulmonary function: the MORGEN study. Thorax.

[32] Tabak C, Arts IC, Smit HA, Heederik D, Kromhout D (2001). Chronic obstructive pulmonary disease and intake of catechins, flavonols, and flavones: the MORGEN study. Am J Respir Crit Care Med.

[33] Walda IC, Tabak C, Smit HA, Räsänen L, Fidanza F, Menotti A (2002). Diet and 20-year chronic obstructive pulmonary disease mortality in middle-aged men from three European countries. Eur J Clin Nutr.

[34] Kowalski M, Kowalska E, Split M, Split W, Pawlaicki L, Kowalski J (2005). Assessment of oral cavity mucosa and teeth state in patients with chronic obstructive pulmonary disease- part 1. Pol Merkur Lekarski.

[35] Grönberg AM, Slinde F, Engström P, Hulten L, Larsson S (2005). Dietary problems in patients with severe chronic obstructive disease. J Hum Nutr Diet.

[36] Gibson RS (2005). Principles of Nutritional Assessment.

[37] Vorrink SNW, Kort HSM, Troosters T, Lammers JWJ (2011). Level of daily physical activity in individuals with COPD compared with healthy controls. Respir Res.

[38] Kobayashi A, Yoneda T, Yoshikawa M, Ikuno M, Takenaka H, Fukuoka A (2000). The relation of fat-free mass to maximum exercise performance in patients with chronic obstructive pulmonary disease. Lung.

[39] Andersson M, Slinde F, Grönberg AM, Svantesson U, Janson C, Emtner M (2013). Physical activity level and its clinical correlates in chronic obstructive pulmonary disease: a cross-sectional study. Respir Res.

[40] Baumgartner RN, Koehler KM, Romero L, Garry PJ (1996). Serum albumin is associated with skeletal muscle in elderly men and women. Am J Clin Nutr.

[41] Cesari M, Penninx BW, Lauretani F, Russo CR, Carter C, Bandinelli S (2004). Hemoglobin Levels and Skeletal Muscle: results from the InCHIANTI Study. J Gerontol A Biol Sci Med Sci.

[42] Bingham SA, Luben R, Welch A, Wareham N, Khaw KT, Day N (2003). Are imprecise methods obscuring a relation between fat and breast cancer?. Lancet.

[43] Crozier SR, Inskip HM, Godfrey KM, Robinson SM (2008). Dietary patterns in pregnant women: a comparison of food-frequency questionnaires and 4 d prospective diaries. Br J Nutr.

